# Enzymatic hydrolysate from velvet antler suppresses adipogenesis in 3T3-L1 cells and attenuates obesity in high-fat diet-fed mice

**DOI:** 10.17179/excli2016-638

**Published:** 2017-03-22

**Authors:** Yuling Ding, Yanmei Wang, Byong-Tae Jeon, Sang-Ho Moon, Seung-Hong Lee

**Affiliations:** 1 Department of Animal Bio and Applied Chemistry, Konkuk University, Chungju, 27478, Republic of Korea; 2 Jilin Sino-Rok Institue of Animal Science, Changchun, 130-600, China; 3 Division of Food Bioscience and Korea Nokyong Research Center, Konkuk University, Chungju, 27478, Republic of Korea

**Keywords:** velvet antler, enzymatic hydrolysate, 3T3-L1 cells, high-fat diet, anti-obesity effect

## Abstract

The purpose of the current study was to investigate the potential anti-obesity activity of an enzymatic hydrolysate of velvet antler in inhibiting adipogenesis in 3T3-L1 cells and in high-fat diet (HFD)-fed obese mice. The enzymatic hydrolysate was prepared using the commercial food grade protease, Protamex. The velvet antler Protamex hydrolysate (VAPH) indicated profound inhibitory effects on adipogenesis dose-dependently by decreasing the accumulation of triglycerides and down-regulating expression levels of adipogenesis-related proteins C/EBPα, SREBP-1, and PPARγ. In a mouse model of HFD-induced obesity, oral administration of VAPH (100 and 300 mg/kg for 13 weeks) significantly reduced the body weight gain that had resulted from the HFD. VAPH treatment also lowered the serum glucose and triglyceride levels, while increasing the HDL-C level. Furthermore, the treatment greatly reduced hepatic lipid droplet accumulation as well as the size of adipocytes. Current findings demonstrate that VAPH has profound anti-obesity effects and could be an effective candidate for preventing obesity and obesity-related chronic diseases.

## Introduction

Obesity is defined as gaining excessive body weight in the form of fat, and is a major risk factor for chronic human diseases, such as type 2 diabetes, hypertension, and cardiovascular disease (Xavier and Sunyer, 2002[34]; Lee et al., 2005[22]; Kong et al., 2009[18]). With the incidence of obesity increasing worldwide, the condition has become a serious health issue. Obesity prevention and treatment are important for reducing the risk of obesity-derived diseases. In recent years, there has been increasing awareness of the benefits of health foods from natural products and interest in discovering new phytochemical compounds for human use. Many studies have identified anti-obesity agents from natural products, especially from herbs and plants (Kong et al., 2009[18]; Popovich et al., 2010[26]; Ko et al., 2013[17]; Mohamed et al., 2014[24]). However, despite the discoveries of various bioactive components in animal tissues that can be used in the prevention and treatment of many human diseases (Chen et al., 2010[2]; Ge et al., 2012[6]; Kim et al., 2014[14]; Lee et al., 2015[21]), reports that document the anti-obesity activity of health foods from animal sources are still relatively rare. 

Velvet antler is a typical traditional medicine of animal origin that has been used for over 2000 years and is still recognized in pharmacopeias of Korea, China, and Japan. In places such as East Asia, New Zealand, Canada, and USA, it is also used as a health food supplement and a nutraceutical to prevent diseases, presumably through the actions of its diverse biological components (Sui et al., 2014[30]). Velvet antler is considered to have various pharmacological effects, including stimulation of the immune system, and enhancements of physical strength and sexual function (Kawtikwar et al., 2010[12]; Gilbey and Perezgonzalez, 2012[7]). A previous study has reported improved lipid metabolism in rats administered a water-soluble extract of velvet antler (Cui et al., 2008[3]). However, the effects of velvet antler on obesity-related adipogenesis are unknown. 

Therefore, the purpose of this study was to investigate the effects of an enzymatic hydrolysate of velvet antler on lipid accumulation, adipocyte differentiation, and adipogenesis-related protein expression in 3T3-L1 cells. Moreover, we examined the effect of the hydrolysate on obesity by evaluating several related parameters and histology in high-fat diet-fed obese mice. 

## Material and Methods

### Chemicals

Mouse 3T3-L1 preadipocytes were obtained from the American Type Culture Collection (ATCC, Rockville, MD, USA). Dulbecco's modified Eagle's medium (DMEM), fetal bovine serum (FBS), bovine serum (BS), and penicillin-streptomycin (PS) were purchased from Gibco BRL (Grand Island, NY, USA). Antibodies to peroxisome proliferator activated receptor gamma (PPARγ) and CCAAT/enhancer binding protein (C/EBPα) were obtained from Cell Signaling Technology (Bedford, MA, USA). Antibodies to sterol regulatory element binding protein 1 (SREBP-1) and glyceraldehyde 3-phosphate dehydrogenase were from Santa Cruz Biotechnology (Santa Cruz, CA, USA). 3-Isobutyl-1-methylxanthine (IBMX), dexamethasone, and insulin were obtained from Sigma (St. Louis, MO, USA). All chemicals and reagents used were of analytical grade and obtained from commercial sources. 

### Preparation of velvet antler sample

The velvet antler was kindly provided by the Daesungsan Deer Farm (Daegwallyeong, Korea). It was obtained from a farmed elk at 75 days after casting. The fresh velvet antler was immediately sliced using a bone slicer and frozen at -20 °C. The frozen slices were dehydrated by lyophilization (freeze dryer; Ilshin Lab Co., Ltd, Gyeonggi, Korea) and then ground into a fine powder using a grinder (HMF-3100S; Hanil Co., Gyeongbukgong, Korea).

### Preparation of enzymatic hydrolysate from velvet antler 

Enzymatic hydrolysis of the velvet antler was performed according to a previously reported method (Ko et al., 2013[16]). The ground dried velvet antler powder (6 g) was first homogenized with 600 mL of distilled water (pH 6.0), and 60 μL of Protamex (Novo Nordisk, Bagsvaerd, Denmark) was then added. Enzymatic hydrolysis was conducted at 40 °C for 24 h, after which the hydrolysate was boiled for 10 min at 100 °C to inactivate the enzyme. The hydrolysate was clarified by centrifugation at 3000 × g for 20 min to remove any unhydrolyzed residue. The supernatant of the velvet antler Protamex hydrolysate (VAPH) was filtered, adjusted to pH 7.0, and stored for subsequent use in experiments.

### Analysis of bioactive components

The protein content was determined by using a commercial assay kit (BCA Protein Assay Kit; Thermo Fisher Scientific, Waltham, MA, USA). The sulfated-glycosaminoglycan (sulfated-GAG) content was determined by the dimethylmethylene blue (DMB) dye assay method (Farndale et al., 1982[5]). In brief, the color reagent was prepared by dissolving 0.008 g of DMB in a solution containing 1.185 g of NaCl, 1.520 g of glycine, and 0.47 mL of 12 M HCl dissolved in 500 mL of distilled water. Each sample was mixed with 1 mL of this color reagent, and the absorbance was determined immediately at 525 nm using a spectrophotometer. 

The uronic acid content was determined from the carbazole reaction (Cesaretti et al., 2003[1]). A 50 μL aliquot of standard (carbazole) or samples was placed in a 96-well plate and supplemented with 200 μL of 25 mM sodium tetraborate solution in sulfuric acid. The plate was heated for 10 min at 100 °C in an oven. After cooling the plate at room temperature for 15 min, 50 μL of 0.125 % carbazole dissolved in absolute ethanol was carefully added. Following a repeated round of heating at 100 °C for 10 min in the oven and cooling at room temperature for 15 min, the plate was read using a microtiter plate reader at a wavelength of 550 nm. 

The sialic acid content was determined by the method of Warren (1959[32]) with a slight modification. In brief, samples were hydrolyzed in 0.1 M H_2_SO_4_ in a final volume of 1 mL for 1 h at 80 °C. Both the standard and samples were incubated respectively with 1 mL of periodate solution at 37 °C for 30 min. Then, after the addition of 0.25 mL of 0.32 M sodium thiosulfate solution, the tubes were shaken until the characteristic yellow-brown color disappeared. The reaction was completed by the addition of 1.25 mL of 0.1 M thiobarbituric acid solution and the tubes were then subjected to a repeated cycle of heating to 100 °C for 15 min and cooling to room temperature. The product was extracted with acidic butanol and the optical density was determined at 549 nm. 

### Cell culture and adipocyte differentiation

3T3-L1 preadipocytes were cultured in DMEM containing 10 % BS and 1 % PS at 37 °C under a 5 % CO_2 _atmosphere. To induce differentiation, 2-day post-confluent preadipocytes (designated day 0) were cultured in MDI differentiation medium (DMEM containing 1 % PS, 10 % FBS, 0.5 mM IBMX, 0.25 μM dexamethasone, and 5 μg/mL insulin) for 2 days. The cells were then cultured for another 2 days in DMEM containing 1 % PS, 10 % FBS, and 5 μg/mL insulin. Thereafter, the cells were maintained in post-differentiation medium (DMEM containing 1 % PS and 10 % FBS), with replacement of the medium on every 2 days. To examine the effects of VAPH on the differentiation of preadipocytes to adipocytes, the hydrolysate was added to cells cultured in MDI. Differentiation, as measured by the expression of adipogenic markers and the appearance of lipid droplets, was completed on day 8. The toxicity of VAPH to 3T3-L1 cells was evaluated by MTT assay. No significant toxic effect was observed on the cells treated with VAPH up to a concentration of 150 μg/mL (data not shown); therefore, VAPH amounts up to this concentration were chosen for the experiments. 

### Determination of lipid accumulation by Oil Red O staining 

Cells were seeded in 6-well plates and adipocyte differentiation was induced for 8 days as described in Cell culture and adipocyte differentiation section. On day 8, the cells were stained with Oil Red O (an indicator of cell lipid content) according to a previously described method, with slight modifications (Ramirez-Zacarias et al., 1992[28]). In brief, cells were washed with phosphate-buffered saline (PBS), fixed with 10 % buffered formalin, and stained with Oil Red O solution (0.7 g in 200 mL of isopropanol) for 1 h at room temperature. Thereafter, the Oil Red O staining solution was removed and the plates were rinsed with distilled water and dried. Images of the stained lipid droplets were taken under a light microscope (Olympus D970; Olympus Optical Co., Tokyo, Japan). Finally, the dye retained in the cells was eluted with isopropanol and quantified by measuring the optical absorbance at 520 nm.

### Measurement of triglyceride content 

Cellular triglyceride contents were determined using a commercial triglyceride colorimetric assay kit (Cayman Chemical, Ann Arbor, MI, USA) according to the manufacturer's instructions. The cells were washed twice with PBS, scraped into a homogenizing solution, and sonicated to homogenize the cell suspension. The residual cell lysate was centrifuged at 3000 × g for 5 min at 4 °C to remove the fat layer. The supernatants were analyzed for the content of triglycerides and proteins. Triglycerides were normalized with the respective protein concentration, employing bovine serum albumin as the calibration standard. Results are represented as the amount of triglyceride in milligram to an equivalent of cellular proteins (in milligram).

### Western blot analysis

Cell lysates were prepared using lysis buffer containing 20 mM Tris, 10 mM Na_4_P_2_O_7_, 5 mM ethylenediaminetetraacetic acid, 100 mM NaF, 2 mM Na_3_VO_4_, 10 mg/ mL aprotinin, 1 % NP-40, 1 mM phenylmethylsulfonyl fluoride, and 10 mg/mL leupeptin. Cellular debris was removed by centrifugation at 10000 × g under 0 °C for 15 min. The protein concentrations were analyzed using the BCA Protein Assay Kit. The protein levels were normalized to 50 μg for the electrophoresis on a sodium dodecyl sulfate polyacrylamide gel (SDS-PAGE). The separated protein bands were transferred onto a nitrocellulose membrane. The nonspecific binding sites were blocked with 5 % non-fat dry milk in TBST (25 mM Tris-HCl, 2.65 mM KCl, 137 mM NaCl, 0.05 %, Tween 20, pH 7.4). The primary antibodies were introduced at a 1:1000 dilution, following overnight incubation at 4 °C. The membranes were gently rinsed using TBST and incubated with the respective secondary antibodies at a 1:3000 dilution. An ECL Western blotting detection kit was used to develop the signals by exposing on to X-ray films. 

### Animal experiment and study design 

C57BL/6 male mice, 5 weeks old and weighing 19-22 g, were purchased from Jung-Ang Lab Animal Inc. (Seoul, Korea). All mice were housed in a room with controlled temperature (22 °C), humidity (55 %), and lighting (12 h light/dark cycle), and given water ad libitum. After acclimatization with a pelletized commercial diet for 1 week, all the experimental mice were divided randomly into four groups (n = 6/group): normal diet (ND), high-fat diet (HFD) control, HFD+VAPH100 (100 mg/kg of VAPH), and HFD+VAPH300 (300 mg/kg of VAPH). The ND group was fed a normal diet, whereas the HFD, HFD+VAPH100, and HFD+VAPH300 groups were fed a diet high in fat [45 % (w/w) fat; Jung-Ang Lab Animal Inc]. All diets were fed ad libitum. The VAPH sample was dissolved in saline and administered orally to the HFD+VAPH100 and HFD+VAPH300 groups, whereas saline was administered orally to the ND and HFD groups, for 13 weeks. Body weights were measured once every week. After 13 weeks, the mice were anesthetized and blood and organs were collected. 

### Serum biochemical analysis

All mice were fasted for 12 h before sacrifice. Blood was collected and centrifuged at 3000 × g for 10 min at 4 °C to obtain the serum. The total cholesterol (TC), high-density lipoprotein cholesterol (HDL-C), glucose, and triglyceride (TG) levels were determined by enzymatic methods using commercial assay kits (Asan Pharm. Co., Ltd., Seoul, Korea). 

### Histological analysis of liver and adipose tissues

The epididymal adipose tissues and liver tissues were fixed in a buffer solution containing 10 % formalin and embedded in paraffin. Sections (3 μm thick) were peeled and mounted on glass slides. Xylene and alcohol were used to remove paraffin. Hematoxylin and eosin (H&E) were used to stain the sections. After alcohol dehydration, the photographs of the slides were taken by using a light microscope (Olympus D970; Olympus Optical Co.). 

### Statistical analysis

Data are expressed as mean ± SE. For the *in vitro* studies, differences between the control and treated groups were evaluated by Student's t-test. For *in vivo* studies, differences between the means of the individual groups were assessed by one-way analysis of variance with Duncan's multiple-range tests. For all analyses, a probability value of p < 0.05 was considered statistically significant. All statistical analyses were performed using SPSS software.

## Results

### Main ingredients of VAPH

The main ingredients of VAPH are shown in Table 1(Tab. 1), where the total extraction yield was 35.33 %. The protein content was 348.25 mg/g and was, therefore, higher than the concentrations of the other extractable components. The other main component was uronic acid (198.59 mg/g), whereas sulfated-GAGs (33.13 mg/g) and sialic acid (5.55 mg/ g) were in minor amounts. These results suggest that the protein of VAPH were major bioactive hydrolysate.

### Effect of VAPH on intracellular lipid accumulation in adipocytes 

**Figure 1:**
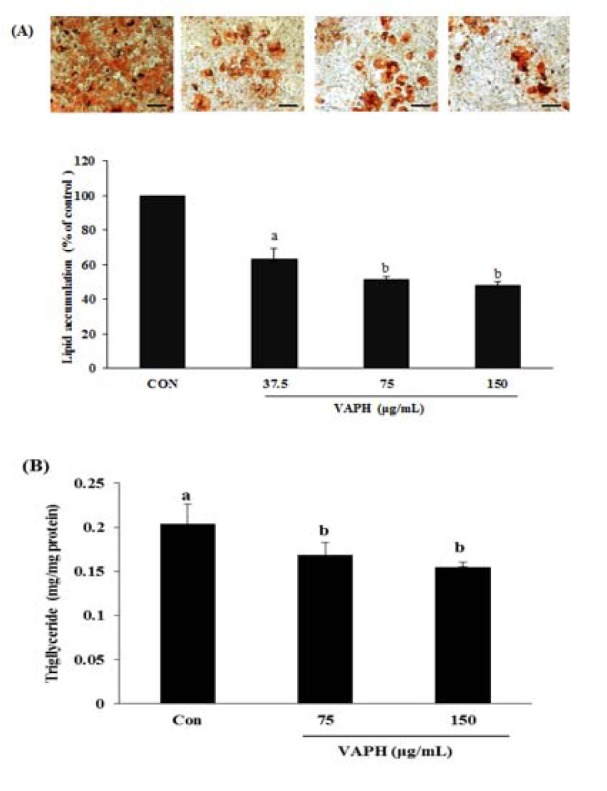
(A) Intracellular lipid accumulation in 3T3-L1 adipocytes after completion of the differentiation process. Microscopic images of adipocytes stained with Oil Red O, and quantification of the lipid accumulation in VAPH-treated and non-treated (control) adipocytes after Oil Red O elution. (B) Effect of VAPH on triglyceride levels in 3T3-L1 adipocytes. The values are expressed as milligram of triglyceride per milligram of cellular protein. Each value is expressed as mean ± SE (n = 3). The letters a and b indicate that the means are significantly different (p < 0.05). Scale bar, 50 μm. VAPH, velvet antler Protamex hydrolysate.

Anti-adipogenic effects of VAPH on the differentiation of preadipocyte into adipocytes was examined by using 3T3-L1 preadipocytes exposed to VAPH for 8 days (from days 0 to 8). Lipid accumulation was taken as a measurement of adipogenesis, and quantified at the end of the proposed differentiation period by assessing the triglyceride levels and Oil-Red-O staining. As shown in Figure 1A(Fig. 1), VAPH significantly reduced the intracellular lipid accumulation in differentiated adipocytes in a dose-dependent manner. Furthermore, VAPH significantly decreased the intracellular triglyceride levels relative to that in untreated control cells at all VAPH concentrations tested (Figure 1B(Fig. 1)). These results indicate that VAPH effectively blocks the formation of mature 3T3-L1 adipocytes. 

### Effect of VAPH on the expression of adipogenic-specific proteins

**Figure 2:**
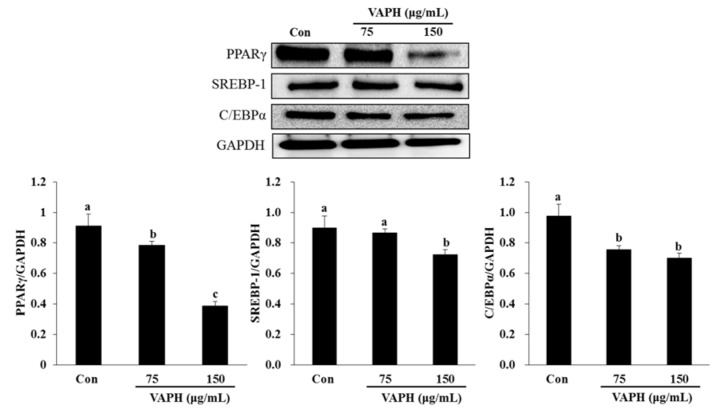
Effect of VAPH on SREBP-1, PPARγ, and C/EBPα expression in 3T3-L1 adipocytes, as examined by Western blot analysis. Each value is expressed as mean ± SE (n = 3). The letters a, b, and c indicate that the means are significantly different (p < 0.05). C/EBPα, CCAAT/enhancer binding protein alpha; GAPDH, glyceraldehyde 3-phosphate dehydrogenase; PPARγ, peroxisome proliferator activated receptor gamma; SREBP-1, sterol regulatory element binding protein 1; VAPH, velvet antler Protamex hydrolysate

To elucidate the molecular mechanisms by which VAPH inhibits adipogenesis, its effect on the protein expression of the adipogenic transcription factors PPARγ, SREBP-1, and C/EBPα was investigated using Western blot analysis (Figure 2(Fig. 2)). As expected, the results showed that the presence of VAPH significantly reduced the protein expression levels of these three transcription factors, especially that of PPARγ (Figure 2(Fig. 2)). These data suggest that VAPH results in the down-regulated expression of adipogenesis transcription factors, with the subsequent inhibition of adipocyte differentiation. 

### Effect of VAPH on HFD-induced obesity in vivo

**Figure 3:**
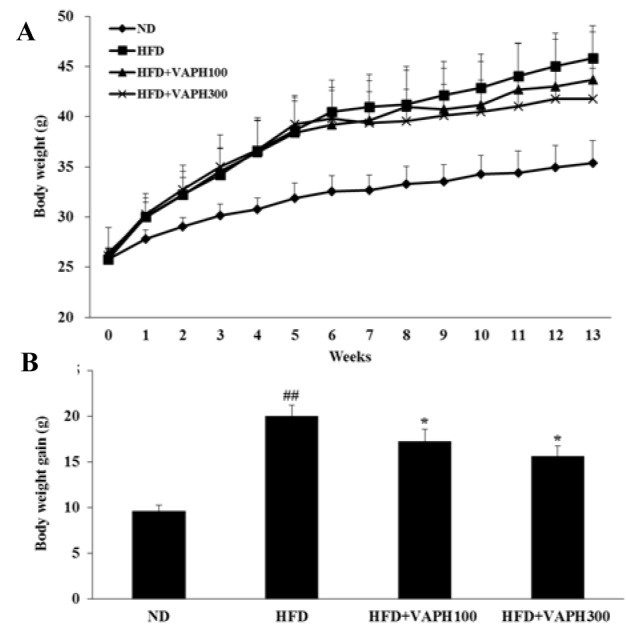
Effects of VAPH supplementation on changes in body weight (A) and body weight gain (B) in HFD-fed obese mice. The values are expressed as mean ± SE (n = 6). ^##^p < 0.005 compared with the ND group, *p < 0.05 compared with the HFD group. ND, normal diet; HFD, high-fat diet-fed control; HFD+VAPH100, HFD and 100 mg/kg of velvet antler Protamex hydrolysate; HFD+VAPH300, HFD and 300 mg/kg of velvet antler Protamex hydrolysate

The effect of VAPH on obesity was investigated using HFD-fed C57BL/6 male mice. The body weight changes during the experiment are shown in Figure 3A(Fig. 3). During the study duration spanning 13-weeks, the mice in the HFD group indicated increased body weight significantly from the starting weight, compared to the ND group. Yet, the body weights of HFD+VAPH100 and HFD+VAPH300 groups were lower relative to the HFD group. After the 9^th^ week, a significant difference between the body weight of the HFD and HFD+VAPH groups were observed upon VAPH treatment. By the end of the experimental period, the body weight gain in VAPH-treated groups significantly attenuated in comparison to the HFD group (Figure 3B(Fig. 3)).

### Effect of VAPH on serum biochemical levels 

**Figure 4:**
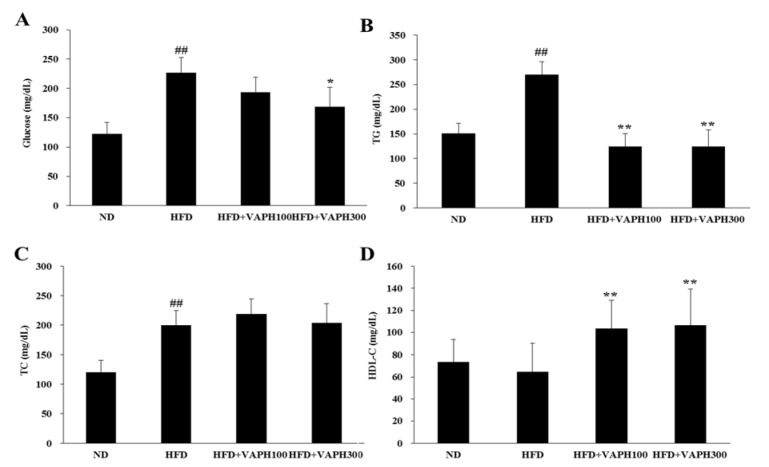
Effects of VAPH supplementation on serum glucose (A), triglyceride (B), total cholesterol (C) and high-density lipoprotein cholesterol (D) levels in HFD-induced obese mice. The values are expressed as mean ± SE (n = 6). ^##^p < 0.005 compared with the ND group, *p < 0.05 compared with the HFD group, and **p < 0.005 compared with the HFD group. ND, normal diet; HFD, high-fat diet-fed control; HFD+VAPH100, HFD and 100 mg/kg of velvet antler Protamex hydrolysate; HFD+VAPH300, HFD and 300 mg/kg of velvet antler Protamex hydrolysate

The serum biochemical levels are shown in Figure 4(Fig. 4). For 13 weeks, the serum glucose and TG levels were significantly higher in the HFD group than in the ND group. However, the TG levels were significantly lower in both VAPH-treated groups than in the HFD group (Figure 4B(Fig. 4)). Furthermore, the glucose levels of the HFD+VAPH300-treated mice were significantly lower than those of the HFD-treated mice (Figure 4A(Fig. 4)). The TC levels were significantly increased in the HFD group, compared with the ND group. However, no significant difference was observed between the HFD and HFD+VAPH groups (Figure 4C(Fig. 4)). Although the HDL-C levels were not significantly different between the HFD and ND groups, the HFD+VAPH groups had significantly higher levels than the HFD group (Figure 4D(Fig. 4)).

### Effect of VAPH on histopathological changes in obese mice liver and adipose tissues

**Figure 5:**
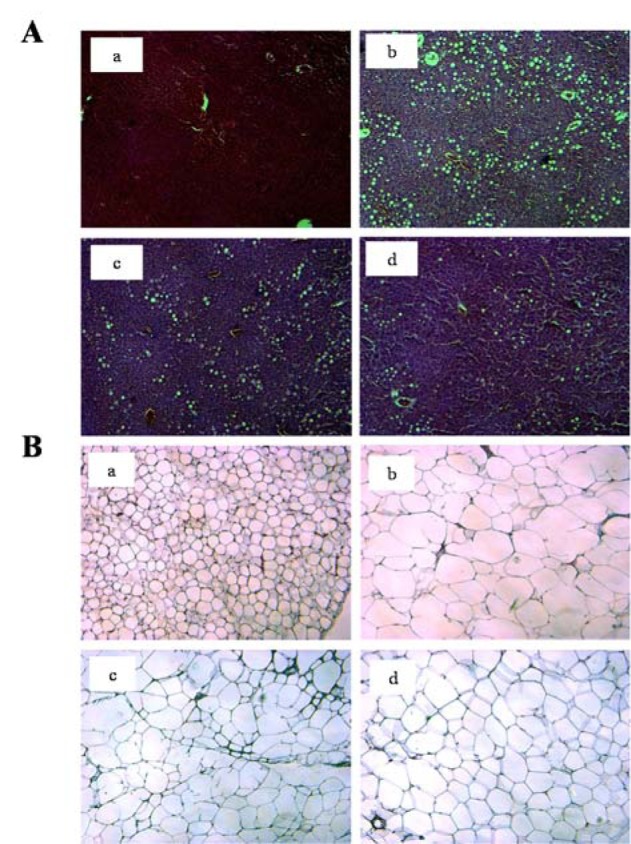
Histopathological changes in liver and adipose tissues. (A) Accumulation of lipid droplets in liver tissue. (B) Changes in the size of adipocytes in epididymal white adipose tissue. Mice were assigned to one of four groups (a: normal diet group; b: high-fat diet-fed (HFD) group; c: HFD plus 100 mg/kg body weight (BW) of velvet antler Protamex hydrolysate (VAPH) group; and d: HFD plus 300 mg/ kg BW of VAPH group) and treated for 13 weeks. The mice were sacrificed and liver and white adipose tissue samples were obtained, fixed, and sliced. The sections were stained with hematoxylin and eosin and examined under a light microscope (125× magnification).

H&E stains were used to stain adipose and liver tissues for the histochemical observation. The HFD group (Figure 5A(Fig. 5), panel b) indicated a substantial accumulation of hepatic lipid droplets, compared to the ND group (Figure 5A(Fig. 5), panel a), whereas it indicated a reduced accumulation in groups that received VAPH treatment (Figure 5A(Fig. 5), panels c and d). In particular, droplet accumulation in the HFD+VAPH300 group was almost completely reduced to that of the ND group level. As shown in Figure 5B(Fig. 5), the size of adipocytes in epididymal tissue was much larger in the HFD group (panel b) than in the ND group (panel a). However, the size of adipocytes in the VAPH-treated groups was dramatically smaller than that of the HFD group (panels c and d). These results show that VAPH efficiently prevents fat accumulation in the liver and the increase in adipocyte size. 

## Discussion

Velvet antler is a typical traditional animal medicine that has been used for over 2000 years. It has been used in traditional Korean and Chinese medicines to prevent and treat many human diseases (Sui et al., 2014[30]; Lee et al., 2015[21]). However, the anti-obesity effect of velvet antler has not been studied. Obesity is a major health issue throughout the globe and an issue with increased concern in the 21st century (Omoleke, 2011[25]). It is associated with the progression of metabolic disease conditions such as type 2 diabetes, hypertension and cardiovascular disorders (Xavier and Sunyer, 2002[34]; Lee et al., 2005[22]; Kong et al., 2009[18]). Many recent studies report anti-obesity agents from natural products (Kong et al., 2009[18]; Popovich et al., 2010[26]; Ko et al., 2013[17]; Mohamed et al., 2014[24]), and interest in exploring the applications of medicinally beneficial products has increased (Kessler et al., 2001[13]). However, reports that document the anti-obesity activity of health foods containing unique animal ingredients are still relatively rare. Therefore, this study is the first to investigate the anti-obesity effect of an enzymatic hydrolysate from velvet antler *in vitro* and *in vivo*, using 3T3- L1 cells and mice fed a high-fat diet, respectively. 

The traditional extraction of bioactive components from velvet antler is generally done via simmering in hot water. However, there is some controversy surrounding this approach, due largely to the extremely limited recovery of water-soluble components in water extractions. During the last decade, enzyme-assistant extraction has successfully been applied for the successful extraction of numerous biologically active natural products from a wide variety of organisms. This technique results in higher yields of bioactive compounds that also show enhanced biological activity compared with ingredients extracted by water or organic solvents employing harsh conditions (Lee et al., 2012[20]; Puri et al., 2012[27]; Ko et al., 2013[16]; Lee et al., 2015[21]). Therefore, in the present study, the velvet antler was enzymatically hydrolyzed using the commercial protease Protamex to make it acceptable as a health food and nutraceutical. It has been reported that velvet antlers are rich in polypeptides and proteins, which are considered to be the most prominent bioactive components (Sui et al., 2014[30]). In this study, the protein content in VAPH was higher than that of the other extractable components. VAPH also contained a significant quantity of uronic acid, along with minor amounts of sulfated-GAGs and sialic acid. These results suggest that the main ingredients of VAPH are protein and uronic acid (an acidic polysaccharide). The sialic acid content of the VAPH obtained in this study was similar to that observed by Je et al. (2010[9]), who used hot water extracts. However, the sulfated-GAG and uronic acid contents in VAPH were higher than those from the hot water extracts. Taken together, these data indicate that enzymatic extraction techniques for obtaining the bioactive components of velvet antler may be more advantageous than hot water extraction.

Possible mechanisms for reducing obesity include decreasing the energy or food intake, increasing energy expenditure, and inhibiting preadipocyte differentiation, lipogenesis, and proliferation (Wang and Jones, 2004[31]). Differentiation of preadipocyte into mature adipocytes and accumulation of fat play key roles in the pathogenesis of obesity (Jeon et al., 2004[10]). In this study, the effect of VAPH on preadipocyte differentiation was investigated by measuring lipid accumulation as well as the expression of several adipogenesis-related proteins in differentiated 3T3-L1 cells. VAPH treatment dose dependently reduced the triglyceride levels along with the absorbance values of Oil Red O solution leached into the cytoplasm of treated cells, suggesting that the function of hydrolysate proceeds by inhibiting adipogenesis through reducing lipid accumulation. The present study is the first to demonstrate the effectiveness of VAPH in this respect. 

Adipogenesis is induced through the action of adipogenic transcription factors such as PPARγ, SREBP-1, and C/EBPα (White and Stephens, 2010[33]), which are regulated in the early stage of adipocyte differentiation (Latasa et al., 2000[19]; Luong et al., 2000[23]). Therefore, overexpression of these transcription factors can accelerate adipogenesis. To evaluate whether VAPH possesses the ability to inhibit adipocyte differentiation through PPARγ, SREBP-1, and C/EBPα, the expression of these proteins was measured using Western blot analysis. The PPARγ, SREBP-1, and C/EBPα expression levels were down-regulated in the presence of VAPH compared with control adipocytes, suggesting that the hydrolysate may indeed inhibit lipid accumulation by blocking the expression of these transcription factors.

Although the causes of obesity are complex, the dietary element, especially a diet containing excessive fat, is a known risk factor. Because animals fed a HFD are considered to be suitable models for obesity studies (Kang et al., 2012[11]), we used mice fed a HFD to examine the anti-obesity effects of VAPH *in vivo*. Mice fed a HFD gained significantly more weight than those fed a normal diet, confirming that the diet-induced obesity was successful. However, interestingly, the body weight gain decreased significantly in the HFD+VAPH-treated groups compared with mice fed only a HFD. This supported the results of our *in vitro* study, in that VAPH has an anti-adipogenic effect on preadipocyte differentiation. 

Excessive fat accumulation and weight gain lead to hyperlipidemia and other metabolic disorders related to obesity (Hollie and Hui, 2011[8]). In the present study, the HFD group exhibited significantly higher levels of serum TG, glucose, and TC than the ND group, as expected. However, the HFD groups treated with VAPH demonstrated reversal of the hyperlipidemic blood status, showing decreased serum TG and glucose contents and increased serum HDL-C levels. Previous studies have reported on the effectiveness of velvet antler water-soluble extracts in decreasing the TG levels and increasing the HDL-C levels in rats (Cui et al., 2008[3]), which are similar to our results obtained with VAPH on mice.

We also found that the 13-week VAPH treatment of obese mice resulted in markedly decreased hepatic accumulation of lipid droplets compared with the non-treated obese mice. This suggests that VAPH might improve non-alcoholic fatty liver disease associated with obesity. Likewise, reduced lipid accumulation was seen in the epididymal white adipose tissue of VAPH-treated obese mice relative to the untreated HFD group. 

 The current study had certain limitations mainly the relatively short-term evaluation that spans 13 weeks may not be appropriate in predicting long-term effects of VAPH. Although the current study demonstrates the anti-obesity effect of VAPH, the bioactive component/s responsible for the observed effects remains vague. Some of the bioactive peptides obtained from bioactive hydrolysate containing large amounts of protein have been reported to show anti-adipogenic effects in cell lines (Kim et al., 2007[15]; Delattre et al., 2012[4]; Singh et al., 2014[29]). As VAPH contains a high amount of protein, further studies will be needed to identify them as the bioactive peptides in the hydrolysate. 

In conclusion, these results are the first to demonstrate an inhibitory effect of VAPH on adipogenesis. In addition, VAPH could beneficially reduce the body weight gain and improve the lipid profile in mice fed a high-fat diet. Therefore, we suggest that VAPH may be a promising candidate for preventing obesity or obesity-related diseases. 

## Notes

Sang-Ho Moon and Seung-Hong Lee (Division of Food Bioscience and Korea Nokyong Research Center, Konkuk University, Chungju, 27478, Republic of Korea; Tel.: 82-43-840-3525, E-mail: leesh80@kku.ac.kr) contributed equally as corresponding authors.

## Acknowledgements

This research was supported by Basic Science Research Program through the National Research foundation of Korea (NRF) funded by the Ministry of Education (NRF-2014R1A1A2056551).

## Conflict of interest

The authors declare that they have no conflict of interest.

## Figures and Tables

**Table 1:**
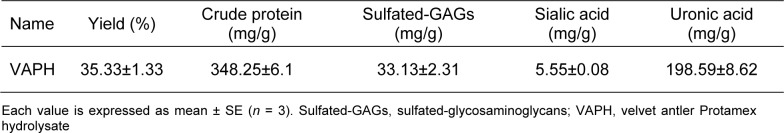
Contents of main ingredients of enzymatic hydrolysates from velvet antler
